# Medical Problems for Public Consumption

**Published:** 1912-06-29

**Authors:** 


### MEDICAL PROBLEMS FOR PUBLIC CONSUMPTION.

Whether or not the appetite of the man in the street for popular explanations of medical mysteries and the inner workings of the doctor's daily round be equal to the fare provided for him, it cannot be gainsaid that there has been a plentiful supply of such literature during the last few years. Quite apart from the interest evoked by the Insurance Act much has been written on the possibilities of a State medical service, the present condition of the Poor-Law doctor's work, and on the promising field of preventive medicine. We need only mention Dr. Benjamin Moore's "Dawn of the Health Age" and Dr. Mackenzie's "Health and Disease" as fair samples of this class of literature. Now our attention is claimed by an experienced general practitioner, Dr. H. de Carle Woodcock, who provides a series of vigorous essays under the title of "The Doctor and the People." Medical men in general practice will appreciate Dr. Woodcock's crisp and vivid descriptions and the man in the street will enjoy his often telling *obiter dicta*. Here we have a readable example of the combination of the philosophy of the general with the description of the particular. The book attempts to cover a wide field and touches—sometimes very aptly—on almost all those problems which confront the medical man from the time he enters as a student till he retires from active work, whether in town or country practice, in surgery or in consulting medicine, in dispensary or in public-health work. Throughout we are forced to realise once more with what halting footsteps the road to prevention has been followed. His chapter on "Poor-Law Experiences" is a vivid exercise in realism, and his portrayal of the horrors he has witnessed is an outstanding feature of the book.

Among other suggestions for improving the state of Poor-Law medical relief we note that the author inclines to making such appointments part-time ones (except, of course, the resident posts) as he believes that private practice increases the keenness of medical men for their work—an opinion which will, we believe, be generally agreed to. On the subject of hospitals in general Dr. Woodcock has a chapter full of shrewd observations which, if they contain nothing new, are none the less worth reiteration. We have again the contrast between the voluntary hospital with the understaffed Poor-Law institution, the picture of the picnics in the out-patient waiting halls, and the problem of the paying patient. The now prominent question of the control of tuberculosis receives a considerable amount of lucid exposition, and these chapters will add much to the interest of the book to the lay reader.

Leaving at last the problems of to-day the author devotes the latter part of his book to lively anecdote and characterisation. Many well-known teachers fortunately still among us figure in the chapter entitled "Lectures and Personalities," while the "Doctors from a Bookshelf" are made to appear before us in an entertaining if not deeply considered array. There is more in this volume from the Midwives Act to the policy of the British Medical Association, but if it lacks exhaustiveness it may yet serve to stimulate inquiry, and that, probably, is what medical authors of this kind of book and the public for which they cater are feeling the desire for at present. It requires very little reflection to see that the medical profession is at a turning point in its history. Like all organisms, it is at the beginning of its periodic transmutation. How this may turn out for medicine and its professors we have not space here to inquire, but for those of our readers who delight in prophecy we may add that the crisis medicine is undergoing at present is one which the lawyers will have to undergo not later than fifty years hence.

